# Opposite Epigenetic Associations With Alcohol Use and Exercise Intervention

**DOI:** 10.3389/fpsyt.2018.00594

**Published:** 2018-11-15

**Authors:** Jiayu Chen, Kent E. Hutchison, Angela D. Bryan, Francesca M. Filbey, Vince D. Calhoun, Eric D. Claus, Dongdong Lin, Jing Sui, Yuhui Du, Jingyu Liu

**Affiliations:** ^1^The Mind Research Network, Albuquerque, NM, United States; ^2^Department of Psychology and Neuroscience, University of Colorado at Boulder, Boulder, CO, United States; ^3^Center for BrainHealth, School of Behavioral and Brain Sciences, University of Texas at Dallas, Dallas, TX, United States; ^4^Department of Electrical Engineering, University of New Mexico, Albuquerque, NM, United States; ^5^Brainnetome Center and National Laboratory of Pattern Recognition, Institute of Automation, Chinese Academy of Sciences, Beijing, China; ^6^School of Computer & Information Technology, Shanxi University, Taiyuan, China

**Keywords:** alcohol, exercise, methylation, impaired control, synaptic plasticity

## Abstract

Alcohol use disorder (AUD) is a devastating public health problem in which both genetic and environmental factors play a role. Growing evidence supports that epigenetic regulation is one major mechanism in neuroadaptation that contributes to development of AUD. Meanwhile, epigenetic patterns can be modified by various stimuli including exercise. Thus, it is an intriguing question whether exercise can lead to methylation changes that are opposite to those related to drinking. We herein conducted a comparative study to explore this issue. Three cohorts were profiled for DNA methylation (DNAm), including a longitudinal exercise intervention cohort (53 healthy participants profiled at baseline and after a 12-months exercise intervention), a cross-sectional case-control cohort (81 hazardous drinkers and 81 healthy controls matched in age and sex), and a cross-sectional binge drinking cohort (281 drinkers). We identified 906 methylation sites showing significant DNAm differences between drinkers and controls in the case-control cohort, as well as, associations with drinking behavior in the drinking cohort. In parallel, 341 sites were identified for significant DNAm alterations between baseline and follow-up in the exercise cohort. Thirty-two sites overlapped between these two set of findings, of which 15 sites showed opposite directions of DNAm associations between exercise and drinking. Annotated genes of these 15 sites were enriched in signaling pathways related to synaptic plasticity. In addition, the identified methylation sites significantly associated with impaired control over drinking, suggesting relevance to neural function. Collectively, the current findings provide preliminary evidence that exercise has the potential to partially reverse DNAm differences associated with drinking at some CpG sites, motivating rigorously designed longitudinal studies to better characterize epigenetic effects with respect to prevention and intervention of AUD.

## Introduction

Alcohol use disorder (AUD) is a devastating public health problem that causes a large economic burden. The lifetime prevalence is estimated to be 17.8% for alcohol abuse and 12.5% for alcohol dependence (1), reflecting the necessity of developing effective prevention or treatment strategies. From the neurobiology perspective, the progression from controlled alcohol use to compulsive and addictive behaviors is hypothesized to be encouraged by reinforcement and neuroadaptation mechanisms, where alcohol exposure elicits neuroadaptive changes which promote drug-related responses (i.e., reinforcement) (2, 3). It has been revealed that the process of developing AUD is largely shaped by genetic and environmental factors that affect the vulnerability to initial use and to shift from use to addiction (4, 5). Family and twin studies estimate the heritability to be 0.5–0.7 for alcohol dependence (6–8) which has been well-acknowledged as a complex polygenic disorder (5, 9). The remaining variability in liability is likely attributable to individual specific environmental factors rather than those shared by families (7).

Hence, it is not surprising that there has been growing interest in exploring epigenetic mechanisms in AUD. Epigenetics integrates genetic and environmental effects (e.g., emotional stressors and social adversities) and can influence biological functions through regulating gene expression (10). Growing evidence supports that epigenetic regulation is one major mechanism through which alcohol consumption and stress result in changes in synaptic systems and neurocircuitry, which contribute to further changes in drinking behavior (11, 12). One commonly studied epigenetic mechanism is DNA methylation (DNAm), which is predominantly found in cytosines of dinucleotide sequence of CpG (10). With respect to AUD, it has been shown in animal studies that alcohol exposure appears to induce transgenerational alterations in DNAm patterns that influence gene expression and neuronal functions in hypothalamus involved in neuroadaptation (13). In humans, altered promotor DNAm levels in peripheral blood have been reported in a variety of genes related to AUD, including dopamine transporter (*DAT*) (14), N-methyl-D-aspartate 2b receptor subtype (*GRIN2B*) (15), nerve growth factor (*NGF*) (16), and μ-opioid receptor (*OPRM1*) (17), suggesting the potential of these DNAm sites in peripheral tissue as biomarkers for AUD. A hypothesis-free epigenome-wide DNAm analysis has provided evidence for calcium signaling and immune system process also being involved in the pathology of alcohol dependence (18). No definitive causal relationship can yet be inferred from the observed associations, nevertheless it is speculated that epigenetics may play both roles of predisposition and response in the model of drug abuse (19).

While epigenetics poses a valuable means to elucidate the interactive effects between genetics and environmental factors on AUD, one particularly interesting angle is its dynamic nature, which may shed light on intervention and treatment. DNAm is shown to be a reversible biological signal through pharmaceutical or behavioral interventions, where DNAm patterns in part determined by maternal behavior could be reversed with cross-fostering or methyl supplementation and could further impact on phenotypic outcome (20–22). Extensive efforts have been devoted to programming DNAm through pharmaceutical actions (23). In addition, there has been converging evidence that, physical exercise, as a well-known non-invasive beneficial stimulus, boosts physical and mental health, including cardiac fitness, immune system, as well as, neuronal and cognitive conditions (24–26). Recently it has been shown that exercise modifies the epigenome-wide DNAm pattern that may be inherited to future generations (27, 28). Notably, one study demonstrates that exercise impacts DNAm and mRNA levels of *BDNF* in the rat hippocampus, suggesting exercise playing some role in transcriptional regulation of synaptic plasticity (29) which is the primary mechanism underlying neuroadaptation (2). Direct evidence of exercise moderating the effect of heavy alcohol consumption on white matter damage has also been documented (30).

Thus, it is an intriguing question whether exercise can be a treatment strategy in a way of reversing epigenetic changes that contribute to development of addiction. While a longitudinal design to compare methylation in drinkers before and after exercise intervention is needed to comprehensively explore this issue, the current study aims to exploit available resources to provide preliminary results for assessing if this topic deserves further attention. We conducted blind epigenome-wide association analyses on three datasets: a longitudinal exercise intervention cohort comparing baseline vs. post-exercise intervention follow-up; a cross-sectional cohort of age- and sex-matched hazardous drinkers vs. controls; and a cross-sectional cohort of binge drinkers only. We first identified methylation sites significantly associated with pre- vs. post-exercise in the exercise intervention cohort. In parallel, we identified methylation sites that not only exhibited significant differences between hazardous drinkers and controls (case-control cohort) but also showed linear associations with drinking behavior (drinking cohort). Then among the markers associated with both exercise and drinking, we focused on those markers showing opposite directions of associations between exercise and drinking, and further explored their multivariate associations with drinking behavior.

## Materials and methods

### Participants

Three cohorts were employed for the comparative analysis. Table 1 provides the study-wise demographics.

**Table 1:** Study-wise demographics.

	**Age (min-max, mean ±SD)**	**Sex (F/M)**	**Race**
**EXERCISE COHORT**			
Exercise (53)	18–44, 28.45 ± 7.94	42/11	Caucasian: 35 African American: 2 Asian American: 7 Hispanic: 6 Native: 3 Multiracial: 0
**CASE-CONTROL COHORT**			
Drinker (81)	21–56, 32.12 ± 10.51	53/28	Caucasian: 40 African American: 1 Asian American: 0 Hispanic: 23 Native: 3 Multiracial: 14
Control (81)	20–56, 32.07 ± 10.66	53/28	Caucasian: 36 African American: 3 Asian American: 23 Hispanic: 7 Native: 0 Multiracial: 12
**DRINKING COHORT**			
Drinker (281)	21–56, 31.80 ± 9.89	86/195	Caucasian: 128 African American: 6 Asian American: 2 Hispanic: 72 Native: 17 Multiracial: 56

#### Longitudinal exercise intervention cohort

This cohort was used to identify methylation sites where significant changes in DNA methylation were noted after exercise intervention compared to baseline. The exercise cohort was a subset of the Colorado STRIDE (COSTRIDE) intervention study, as described in (31, 32). This study was approved by the University of Colorado Human Research Committee, the Scientific Advisory Committee of the University of Colorado General Clinical Research Center, and the University of New Mexico's Human Research Review Committee. All the participants provided written informed consent. In brief, COSTRIDE was a trial that compared an exercise-specific intervention to a general health promotion intervention. The recruiting criteria included: healthy individuals (not taking psychotropic medication, not receiving psychiatric treatment, no diabetes, no history of cardiovascular or respiratory disease); not meeting physically activity guidelines (i.e., 90 min or less of average voluntary physical activity per week over the past 3 months) at the baseline time point; having a body mass index (BMI) within the range of 18 and 37.5; free of nicotine dependence (self-defined). The participants were then instructed to increase their moderate-intensity physical activity to at least 5 days a week for 30 min a day, no dietary regimen enforced. For DNAm study, a saliva sample was collected at two time points: month 1 after all the baseline assessments were completed, and month 12 after the whole trial was completed. A total of 100 participants had good-quality epigenome-wide DNAm data for the baseline point and 64 participants had good-quality epigenome-wide DNAm data for both time points (see Data Preprocessing). We further selected 53 participants that showed enough amount of exercise (self-reported 7-Days Physical Activity Recall of the final month ≥ 30 min) to be analyzed for the impact of chronic moderate exercise on DNAm. Note that exercise intervention was only conducted in this longitudinal cohort of healthy individuals. Other cohorts employed in this study did not participate in the exercise intervention experiment.

#### Cross-sectional case-control cohort

This cohort was used to identify CpG sites exhibiting significant differences in DNA methylation between healthy controls and hazardous drinkers. The hazardous drinkers were a subset of 332 participants enrolled for investigating genetic and neurobiological traits related to drinking, as described in previous works (33–35). The University of New Mexico Human Research Review Committee approved the study and all the participants provided written informed consent. The original recruiting criteria included: drinking at least five times in the past month with at least five (male) or four (female) drinks per drinking occasion; no history of severe alcohol withdrawal, brain-related medical problems, or symptoms of psychosis. And the participants were required to be sober on the day of data collection, having a breath alcohol concentration of 0.00. In addition, the following criteria were used to select hazardous drinkers: having an Alcohol Use Disorders Identification Test (AUDIT) total score > 8 (36) and Fagerstrom Test for Nicotine Dependence (FTND) < 6 (no nicotine dependence). The healthy controls were recruited from two studies: (a) an extension of the genetics of drinking study (33–35); and (b) control comparison of an obesity study (37), both approved by University of New Mexico Human Research Review Committee and all the participants provided written informed consent. The recruiting criteria for healthy controls included: free of nicotine dependence (self-defined, about half participants provided FTND scores to confirm); free of any alcohol use problems and any problem drinking history (occasional alcohol use was allowed, ADUIT scores were available for participants to confirm); free of any chronic disease, any brain injury, and any substance use other than alcohol. After going through the same data preprocessing, 81 hazardous drinkers and 81 controls were matched in age and sex and included for the analysis on DNAm differences.

#### Cross-sectional binge drinking cohort

This cohort was used for methylation-drinking behavior association analysis. It consisted of 281 drinkers, which were also a subset of the aforementioned 332 participants enrolled for investigating genetic and neurobiological traits related to drinking. While no additional filtering was applied, 328 participants had good-quality epigenome-wide DNAm data after quality control, among which 281 participants had complete demographic and behavioral assessment data including the Alcohol Dependence Scale (ADS) (38), the AUDIT (36), and the Impaired Control Scale (ICS) for alcohol (39). A total of 13 representative behavioral measures were included for investigation, as listed in Table 2.

**Table 2:** Behavioral assessments investigated for associations with DNA methylation.

**Assessment**	**Sub-category**	**Description**
ADS	ADS-con	Loss of behavior control
	ADS-obs	Obsessive drinking style
	ADS-per	Psychoperceptual withdrawal
	ADS-phy	Psychophysical withdrawal
	ADS-tot	Total ADS
AUDIT	AUDIT-1	How often do you have a drink containing alcohol?
	AUDIT-2	How many drinks do you have on a typical day when you are drinking?
	AUDIT-3	How often do you have 6 or more drinks on one occasion?
	AUDIT-tot	Total AUDIT score
ICS	ICS-total	Total ICS
	ICS-ac	Attempted control
	ICS-fc	Failed control
	ICS-pc	Perceived control

### Data preprocessing

A detailed description of the profiling, quality control and preprocessing of the methylation data can be found in previous works (32, 33). Briefly, DNA was extracted from saliva for all the participants. Illumina HumanMethylation27 BeadChip (www.illumina.com) was used for DNAm profiling, which covered a total of 27,578 CpG sites. Cell type proportions in saliva were estimated by the algorithm of Houseman et al. (40) using methylation data from buccal epithelial cell (https://www.ncbi.nlm.nih.gov/geo/query/acc.cgi?acc=GSE46573) and other leukocyte cell types from the “minfi” package as the reference (41–43). The estimated cell type proportions were included in the *post-hoc* analyses as covariates. The measurements with beta value detection *p* > 0.05 were set as missing. Samples with ambiguous sex information, or measurement missing rate > 5%, were excluded. CpG sites were excluded if they had missing rate > 5% or overlapped with cross-hybridizing probes. Batch effects were adjusted on each CpG site using regression as in our previous work (44). Finally, to assure that we analyzed true biological signals instead of variance from unknown experimental factors, we estimated the measurement error range based on replication tests of randomly selected samples, and we only considered CpGs with inter-subject variation greater than measurement error. The standard deviation (SD) of replication error was 0.045 for the exercise cohort and 0.06 for the case-control and binge drinking cohorts. To standardize the filtering, we chose to set up the SD threshold based on the larger measurement error which was a more stringent filtering. As a result, CpG sites with SD > 0.06 were retained for further analyses in both studies, leading to 3,205 and 5,153 CpG sites for the exercise and drinking associations, respectively.

Particularly for the longitudinal exercise intervention cohort, considering that the two data collection points spanned ~1 year, and we were not able to effectively quantify and disentangle the aging-related variance from that related to exercise given the available data, we chose to eliminate CpG sites potentially affected by aging. For this purpose, we first excluded 353 age-related CpG sites derived for prediction purpose as provided by Horvath et al. (45). In addition, we examined in the 100 baseline samples with good quality DNAm data whether any other CpG sites might also exhibit significant associations with age. Specifically, we modeled DNAm at each CpG site as a function of age and adjusted for sex and race (dummy-coded). To be conservative, a threshold of *p* < 0.01 (uncorrected) was employed to identify CpG sites likely affected by aging. These sites were then excluded from subsequent analyses, leading to a final set of 3,130 CpG sites assessed for exercise-related changes in methylation. And a total of 2,706 CpG sites overlapped between the 3,130 and 5,153 CpG sites included for exercise and drinking association analysis, respectively.

### Opposite DNAm alterations

We first conducted two lines of analysis separately to locate methylation sites associated with exercise and drinking status using paired *t*-test and regression, respectively, as detailed below.

#### DNAm alteration in exercise

One line of analysis aimed to identify CpG sites that showed different DNAm patterns between pre- and post-exercise time points. For the 3,130 CpG sites retained after data preprocessing, we conducted paired *t*-test (baseline vs. follow-up) on the 53 participants. A significant DNAm alteration due to exercise was determined based on *p* < 0.05 (Bonferroni corrected). For a sanity check, we compared our longitudinal exercise data with a previous work by Zeng et al. (46). While most longitudinal exercise studies focused on adipose or muscle tissues (47), Zeng et al. investigated the effects of 6-months moderate exercise on DNAm of blood in a breast cancer population using the Illumina Methylation27 array, which was expected to be more suitable for comparison with our data in the sense of using peripheral tissue and the same methylation array. We then examined in our data how the DNAm patterns changed at the CpG sites previously highlighted in Zeng et al. (46). See Supplemental for details.

#### DNAm association with drinking

We wanted to compare CpG sites, respectively, associated with exercise and drinking to locate those showing opposite directions of associations. While Liu et al. conducted the largest epigenome-wide association study of methylation in relation to alcohol intake (48), they used DNAm extracted from blood and Illumina HumanMethylation450 for methylation profiling, which were different from the tissue and platform used in the exercise cohort. Therefore, we chose to base the analysis on our own data. We conducted a two-step analysis to identify drinking-related CpG sites: (a) we first used the case-control cohort to identify CpG sites that presented differential methylation patterns between drinkers and non-drinkers, namely the group-difference CpG sites. (b) We then further examined whether the methylation at the group-difference CpG sites showed linear associations with drinking behavior (e.g., dependence scale) in the binge drinker cohort. The second step of verifying linear associations was expected to complement the first step of group difference analysis and provide additional guard against false positives in association with drinking.

In step 1, the case-control cohort consisted of 81 hazardous drinkers and 81 controls matched in age and sex, but not race and other unknown factors. Consequently, we used a regression model to identify CpG sites that significantly differed between the two groups. Specifically, the DNAm at each CpG site was modeled as a function of grouping factor (drinker vs. control) and adjusted for race (5 dummy-coded variables). The test was conducted for each of the 5,153 sites on 162 samples. Significant group-difference CpG sites were determined based on *p* < 0.05 (Bonferroni corrected). Subsequently in step 2, the group-difference CpG sites identified in step 1 were examined for associations with behavioral scales in 281 drinkers. Given that the 13 behavioral scales (as shown in Table 2) were highly correlated, we conducted principal component analysis (PCA) on the behavioral data and assessed the methylation associations with the resulting principal components (PCs) to address collinearity. Specifically the 7 top PCs were included in the regression to capture 90% of the variance in the original data. Then, linear regression was used to evaluate the association between each of the 7 PCs and DNAm at each of the CpG sites identified in step 1, adjusted for age, sex, and race. Considering that step 2 was an additional verification of the Bonferroni corrected group-difference CpGs in relation to drinking, we did not impose correction for multiple comparisons at this step, which was a tradeoff for false negatives. Thus, combining the two steps, CpG sites presenting significant differences (*p* < 0.05, Bonferroni corrected) between drinkers and non-drinkers and linear associations (*p* < 0.05, uncorrected) with drinking behavior scales were considered as associated with drinking and were compared with those identified as associated with exercise for potential overlap.

For a sanity check, we compared the CpG sites identified as drinking-related in the above two-step analysis with those identified in the largest epigenome-wide association study of methylation in relation to alcohol intake by Liu et al. (48). In brief, we examined if any of our findings overlapped with the CpG sites presenting associations (*p* < 1 × 10^−4^, uncorrected, as provided in the supplemental information) with continuous or categorical alcohol phenotype in Liu et al., and if the directions of effect were consistent.

The two sets of CpG sites related to exercise and drinking, respectively, were then compared to locate the overlapping sites significantly altered in both studies. We highlighted those showing opposite directions of alterations, i.e., decreased/increased in follow-up than baseline, and increased/decreased in drinkers than controls, as potential biomarkers for exercise intervention. These identified CpG sites went through the following *post-hoc* analyses. First, we investigated if the observed DNAm differences might be affected by cell type proportions. For this purpose, we conducted a regression analysis rather than paired *t*-test in the exercise cohort where DNAm was modeled as a function of grouping factor (baseline vs. follow-up) and adjusted for cell type proportions. In the drinking cohort, the same regression model was used with cell type proportions included as additional covariates. Second, the identified sites were additionally tested for DNAm differences in a subsample of 29 exercise participants who increased their VO_2_ max (maximal oxygen uptake) at the 12-months follow-up. Compared with self-reports, the objectively measured VO_2_ max more accurately reflects exercise status and this test was expected to help confirm exercise induced the observed DNAm alterations rather than other unknown factors. Third, considering that the drinkers and controls were not matched in race in the above test, the highlighted sites were further assessed in a subset of 52 drinkers and 52 controls that were further matched in race to confirm that the observed DNAm differences were not driven by population structure. Finally, we conducted Ingenuity Pathway Analysis (IPA, www.qiagenbioinformatics.com/products/ingenuity-pathway-analysis) to explore if any signaling pathway might be enriched in our highlighted findings.

### Multivariate assessment of DNAm and behavior association

The identified CpG sites all presented opposite directions of alterations between exercise and drinking and might not be independent to each other. Thus, it would be intriguing to examine if any interaction exists among these CpG sites and how the combined effect of multiple variables at network level associates with behavioral scales. For this purpose, we conducted independent component analysis (ICA) (49, 50) to extract clusters of CpG sites and clusters of behavioral assessments presenting independent instead of uncorrelated (as yielded by principal component analysis) patterns and assessed the inter-modality associations based on the extracted multivariate patterns. Note that although for a normal random variable uncorrelated and independent are equivalent, in this work we do not want to make the limiting assumption that drinking behavior and methylation data are Gaussian. While uncorrelated principal components are adequate for addressing collinearity, independent components extracting non-Gaussian components by exploiting the higher order statistical information in general yield better performance in characterizing underlying data structure and has been shown to capture consistent and meaningful multivariate covarying patterns in various types of data (41, 51, 52). The methylation and behavior input data (**X**_1_ and **X**_2_) were separately decomposed into a linear combination of independent components (**S**_1_ and **S**_2_) by X = AS based on Infomax ICA (49, 50). Then methylation-behavior correlations were evaluated based on component loadings (**A**_1_ and **A**_2_). ICA decomposed variables into components by their contribution to each independent distribution pattern. The component loading largely reflects the covariation pattern of the top variables that have high component scores. To avoid capturing components driven by confounding factors, the DNAm data were first adjusted for age, sex, and race at each CpG site using regression and the residuals were used in the ICA analysis. Finally, we evaluated the association between each pair of methylation and behavior component loadings. Significant associations were determined based on Bonferroni correction for all the tested methylation and behavioral component pairs.

## Results

### DNAm alteration in exercise

Paired *t*-test was conducted for each of the 3,130 methylation sites on 53 participants in the longitudinal exercise cohort. Using a Bonferroni corrected threshold of *p* < 0.05, 341 CpG sites were identified to show significant DNAm alterations between baseline and follow-up measurements. Among these 341 sites, 133 were hypermethylated and 208 were hypomethylated in follow-up. While no data were available for an exact replication of the current findings, for the purpose of sanity check, we compared our observations with a previous work by (46) which investigated the effects of moderate exercise on DNAm of blood in a breast cancer population. Twenty-four out of the 43 genes identified in Zeng et al. had CpG sites presenting epigenome-wide significant DNAm changes in our data. Among these 24 genes, 15 showed DNAm changes consistent with those reported in Zeng et al. Another 5 of the 24 genes had underlying CpG sites presenting both positive and negative DNAm changes, for which we could not determine if our observations echoed those of Zeng et al. as the latter did not provide CpG information. The remaining 4 of the 24 genes showed inconsistent DNAm changes between the two studies. See Supplemental and Table S1 for details.

### DNAm association with drinking

In parallel, methylation sites associated with drinking were identified using the cross-sectional case-control and binge drinking cohorts. Specifically, we first conducted regression analysis for each of the 5,153 methylation sites on 81 drinkers and 81 controls matched in age and sex to detect group-difference CpG sites. We then further examined the group-difference CpG sites for linear associations with 13 behavioral assessments (Table 2) in 281 drinkers of the binge drinking cohort. A total of 906 sites showed significant DNAm differences (*p* < 0.05, Bonferroni corrected) between drinkers and controls, as well as, presented associations (*p* < 0.05, uncorrected) with drinking behavior assessments. In the 906 sites identified in alcohol use, 641 sites were hypomethylated while the remaining 265 sites were hypermethylated in drinkers. Among the 906 drinking-related CpG sites identified in the current study, 15 unique sites overlapped with the CpG sites identified as associated with alcohol intake in the largest epigenome-wide association study (48) and 14 out of these 15 overlapping sites showed consistent directions of effect between DNAm level and alcohol use (Table S2).

A total of 32 sites overlapped between the two sets of DNAm markers associated with exercise and drinking, respectively. For 15 out of these 32 sites, the alterations induced by exercise were in the opposite directions to those observed in drinking. Table 3 provides the detailed information for these 15 markers, including the annotated gene, the significance *p*-values, the mean DNAm level in control vs. drinking participants, as well as, exercise baseline vs. exercise follow-up. And Table S3 summarizes the official full names and brief NCBI summaries of the annotated genes of the 15 CpG sites. One of the 15 sites, cg26825412 (promoter of *SOX18*), was also identified to associate with alcohol intake in the largest epigenome-wide association study (48) and showed consistent effect that decreased DNAm at cg26825412 associates with increased alcohol use (Table S2). The observed DNAm differences at the 15 highlighted CpG sites were not likely confounded by cell type proportions as estimated by the algorithm of Houseman et al. (40), given that consistent and significant differences were observed when cell type proportions were further included as covariates (Tables S4, S5). In addition, in both the assessment with the subsample of 29 exercise participants who increased their maximal oxygen uptake (VO_2_ max), and the assessment with the subsample of 52 drinkers and 52 controls that were further matched in race, all the 15 highlighted sites showed the same directions of significant DNAm differences as observed in the primary analysis (Tables S6, S7), indicating a low possibility that our findings were driven by unknown factors in the exercise cohort or population structure in the case-control cohort. IPA revealed that the annotated genes of these 15 CpG sites were enriched in anandamide degradation, as well as, reelin and Wnt/β-catenin signaling, as shown in Table 4.

**Table 3:** CpG sites identified to show opposite directions of significant alterations between exercise and alcohol use.

**CpG_site**	**Gene**	**Drinking**			**Exercise**		
		**Mean_control**	**Mean_drinker**	***P*-value**	**Mean_baseline**	**Mean_followup**	***P*-value**
cg00510787	C6orf96	0.2395	0.1636	7.59E−06	0.2267	0.3120	4.66E−06
cg00542846	APP	0.2209	0.1363	5.71E−09	0.1540	0.2131	3.67E−06
cg06270401	DYRK4	0.4840	0.4200	8.05E−06	0.4549	0.5179	1.37E−05
cg06415153	PITPNM2	0.5131	0.4328	1.37E−11	0.4302	0.5058	1.73E−07
cg07031532	OAZ2	0.0883	0.1587	4.00E−08	0.1454	0.0848	2.07E−06
cg12286890	XCL2	0.6152	0.7205	2.74E−09	0.6534	0.5369	8.58E−12
cg12671744	FAAH	0.2281	0.2919	2.33E−06	0.2737	0.2076	1.63E−07
cg14760714	RPUSD2	0.2151	0.1421	4.09E−09	0.1482	0.2088	4.37E−07
cg15364618	CIDEB	0.1907	0.2983	1.27E−07	0.2916	0.2500	6.93E−06
cg15679651	MAP4K1	0.3130	0.2544	2.15E−07	0.3477	0.4097	1.14E−05
cg17091851	LOC348174	0.4846	0.5437	5.66E−06	0.5238	0.4697	9.25E−08
cg18241160	CDC2L2	0.5656	0.3396	2.38E−12	0.3923	0.5310	1.10E−06
cg24648715	TCEAL3	0.3423	0.2492	1.65E−13	0.2696	0.3173	1.23E−09
cg24792360	FUCA1	0.4656	0.5297	1.00E−07	0.5060	0.4716	3.03E−07
cg26825412	SOX18	0.6743	0.6061	1.45E−11	0.6602	0.7093	1.56E−06

**Table 4:** Significantly enriched canonical pathways identified by IPA.

**Canonical pathway**	**Molecules**	***P*-value**
Anandamide degradation	FAAH	8.14E−04
Reelin signaling in neurons	MAP4K1, APP	2.24E−03
Wnt/β-catenin	SOX18, MAP4K1	7.41E−03

**Figure 1:**
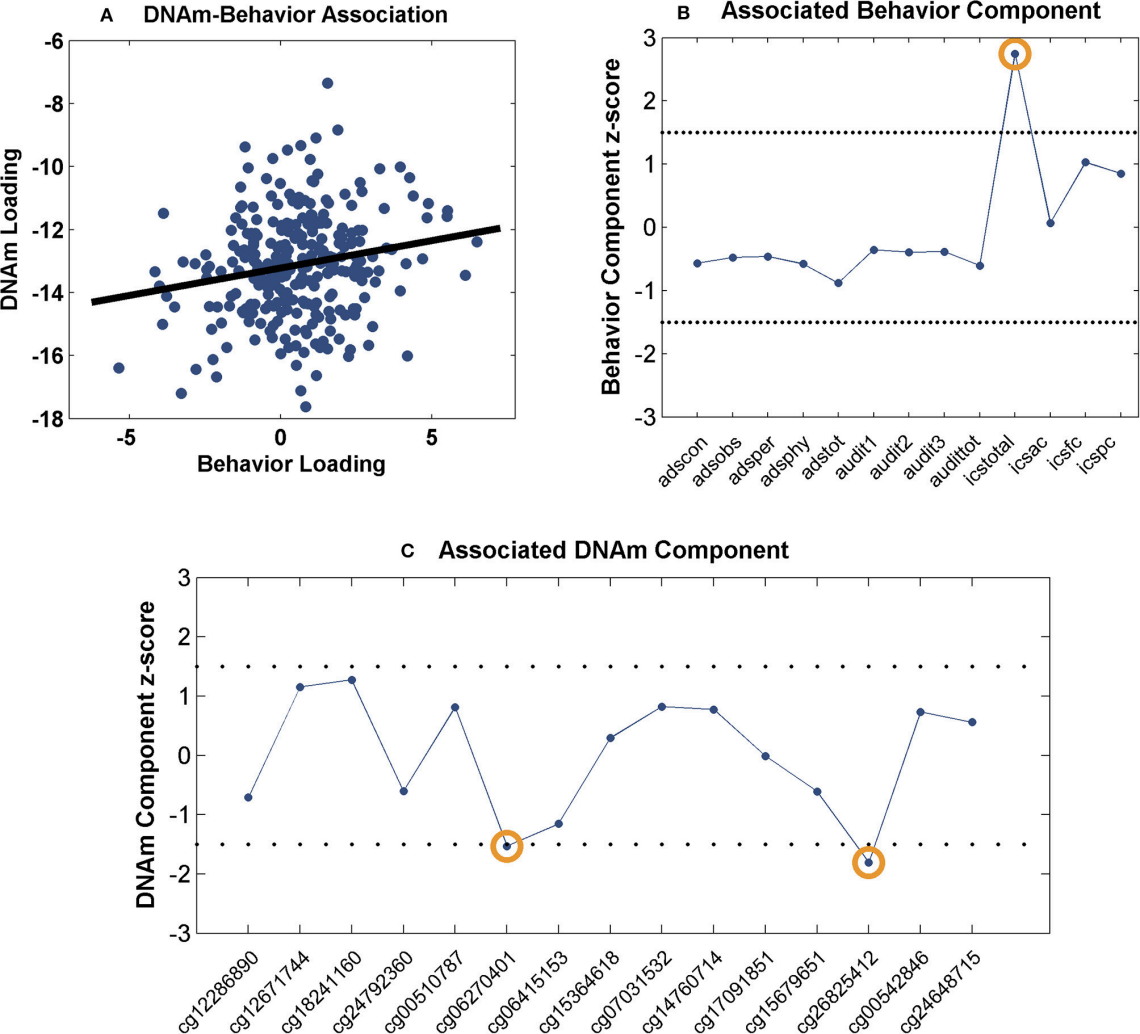
Association between the identified CpG sites and behavioral measures: **(A)** Scatter plot of the methylation and behavior component loadings; **(B)** Scatter plot of the associated behavior component; **(C)** Scatter plot of the associated methylation component. The dashed lines represent threshold of |z-score| > 1.5.

The identified 15 sites were further investigated for network-level associations with 13 drinking behavioral measures (Table 2) in 281 drinkers. The component number was selected to be 2 for the methylation data and 7 for the behavioral data, based on component stability (53). Among the 14 pairs of methylation and behavior components, one pair presented a significant positive correlation (*r* = 0.18, *p* = 3.00 × 10^−3^, passing Bonferroni correction for 14 pairs) as shown in Figure 1A. When thresholded at |z-score| > 1.5, the top contributing behavior measure to the associated component was ICS-total, while the top contributing DNAm sites were cg06270401 (*DYRK4*) and cg26825412 (*SOX18*), as shown in Figures 1B,C. Collectively, the identified association implied that the combined effect of decreased DNAm at cg06270401 and cg26825412 associated with increased ICS-total score.

## Discussion

The epigenome-wide associations of DNAm with alcohol use and exercise were examined. The findings suggest that at specific CpG sites exercise results in changes in methylation levels that are opposite to those associated with alcohol use. Overall the current work provides preliminary molecular evidence for the potential of exercise influencing drinking effect. Future studies that explicitly examine the effect of exercise on drinking through methylation are needed to fully explore these preliminary associations.

The epigenome-wide analysis yielded 906 CpG sites whose methylation levels were significantly associated with drinking status, among which 71% presented decreased DNAm levels in drinkers, echoing the previous epigenome-wide study's finding of a skew toward hypomethylation in alcohol users in peripheral blood (18). On the other hand, the DNAm alterations in exercise appear to be less dominated by one direction. In skeletal muscle, a decrease of global methylation has been noted after acute exercise (54) and most genes were hypermethylated after a 6-months exercise intervention (55). In contrast, the majority of the DNAm alterations were hypermethylation in adipose tissue after a similar 6-months exercise intervention (56). Meanwhile, in another study of sperm DNAm changes after a 3-months exercise training, 43% of the significant alterations were hypermethylation which is comparable to the hypermethylation ratio of 39% observed in the current exercise study. While these observations indicate exercise's impact on DNAm is likely tissue-specific (47), the current study is exempt from the concern of cross-tissue difference given that the opposite alterations of DNAm in exercise and alcohol use were both observed from the saliva tissue.

A total of 15 CpG sites were identified to present opposite directions of alterations between exercise and alcohol use, of which 9 sites were hypomethylated and the remaining 6 sites showed hypermethylation in drinkers. IPA revealed that the annotated genes of these 15 methylation sites were enriched in several pathways, among which the reelin signaling pathway appears to be of particular interest. The reelin pathway is known to play an important role in the formation and maintenance of neural circuits. It regulates neuronal migration and cell positioning during brain development (57) and continues to serve as modulator of synaptic plasticity in adult brain (58), the latter underlying neuroadaptation in the development of addiction (59). As shown in Table 4, two of the identified 15 genes, *APP* and *MAP4K1*, are involved in the reelin pathway. *MAP4K1*, also known as Hematopoietic progenitor kinase 1 (*HPK1*), belongs to the mitogen-activated protein kinase family and functions in T cell receptor signaling and T cell-mediated immune responses (60). Amyloid beta precursor protein (*APP*), while frequently implicated in Alzheimer's disease (AD) and relating to the formation of amyloid plaques (61, 62), may also interact with reelin and modulate synaptic plasticity (63–65). Although the reelin gene (*RELN*) is not highlighted in the current finding (filtered out in preprocessing by the SD threshold), its methylation site cg10007262 shows decreased DNAm in drinkers and increased DNAm after exercise intervention, similar to the observation for cg00542846 (*APP*). In addition, the DNAm levels at these two sites of cg10007262 and cg00542846 are highly correlated in both drinkers and controls, as well as, before and after exercise intervention (see Supplemental for details), which suggests a possibility of a shared regulating mechanism between these two CpG sites. These findings deserve more attention in the future studies of AUD, particularly from the perspective of synaptic plasticity.

Other enriched pathways include anandamide degradation involving the *FAAH* gene, and Wnt/β-catenin signaling involving the *SOX18* and *MAP4K1* genes. The fatty acid amide hydrolase encoded by the *FAAH* gene (cg12671744) is the primary catabolic enzyme for anandamide, which is a fatty acid neurotransmitter belonging to the endocannabinoid system, modulating a variety of cognitive and emotional processes (66) and well-documented for reward and addiction (67). It is noted that, the *FAAH* gene has been found to show higher expression in late-onset AD patients. In the context of AUD, single nucleotide polymorphisms (SNPs) residing in *FAAH* have been associated with problem alcohol use (68, 69). In the current study, drinkers presented hypermethylation at cg12671744, which might associate with downregulation of *FAAH* given that anti-correlation between cg12671744 methylation and *FAAH* mRNA expression has been reported in cancer studies (70). Thus, our observation collectively favors a higher *FAAH* expression for less problematic alcohol use. This observation finds support from animal models which suggests that depletion of the *FAAH* gene leads to increased endocannabinoid signaling that will increase ethanol consumption owing to decreased acute ethanol intoxication (71, 72). Therefore, it is not through cognition that the *FAAH* gene affects alcohol use, which might explain the opposite associations of *FAAH* expression with AD and alcohol use. The full functional impact of cg12671744 methylation and *FAAH* expression awaits further elucidation. The canonical Wnt/β-catenin pathway regulates stabilization of β-catenin that subsequently enters the nucleus and promotes gene expression (73). While this pathway has been widely investigated in cancer studies (73), evidence has also emerged to suggest that the canonical Wnt pathway is a key component in neurodevelopment and thus plays a role in psychiatric diseases (74). Particularly, Cuesta et al. has demonstrated that Wnt/β-catenin pathway contributes to long term neuroadaptations that are necessary for the behavioral response to cocaine, lending support for it playing a role in development of addiction. Meanwhile, the two involved genes, *MAP4K1* and *SOX18*, do not directly interact with Wnt or β-catenin. Instead, *MAP4K1* moderates the signal transduction from transforming growth factor beta receptor (TGFBR) to T-cell factor/lymphoid enhancer-binding factor (TCF/LEF) (75–78), and *SOX18* may interact with CREB-binding protein (CBP) (79) in the Wnt/β-catenin pathway. Overall, it needs to be investigated in greater detail how Wnt/β-catenin signaling relates to AUD and how *SOX18* and *MAP4K1* contribute to this relation.

Two CpG sites were noted for their associations with impaired control over drinking. As shown in Figure 1, a positive correlation was observed between the behavior and methylation loadings, where the top behavioral measure (ICS-total) presented positive component scores and the two top CpG sites presented negative component scores. Collectively, the findings suggest a linear combination of lower DNAm at cg06270401 and cg26825412 associated with higher ICS-total score. Thus, the observed methylation-behavior association is consistent with the observed differential methylation in the two cohorts. Using cg06270401 as an example, given the positive methylation-behavior correlation, the negative methylation component weight and the positive behavior component weight, the lower its DNAm, the higher the ICS-total which indicates more severe impaired control. Meanwhile, this CpG site was hypomethylated in drinkers while exercise intervention increased its DNAm. All these observations favor a higher DNAm at cg062704041. ICS is an instrument for measuring the degree of impairment over control of alcohol consumption shown by a problem drinker. Higher ICS scores indicate increasing degree of impaired control. Individuals with higher ICS scores are less likely to have a successful outcome to treatment and at higher risk of relapse (80). Impaired control affects impulsivity which is a central component in the development of addiction (81). Prolonged alcohol use is known to impair response inhibition (82). On the other side, response inhibition has been shown to moderate the relationship between implicit association and drinking behavior (83), as well as, predict aggregate alcohol-related problems (84). At molecular level, one mechanism underlying inhibition is synaptic potentials generated by GABAergic neurons (85) where μ-opioid receptor (MOP) can serve as a mediator (86, 87). MOP has also been reported to modulate neural activity involved in motivation to drink (88). Interestingly, one of the identified genes, *SOX18* hosting cg26825412, encodes the SRY-box 18 transcription factor that has been found to regulate MOP gene expression in mice (89), presenting a potential pathway that connects *SOX18* with impaired control. *DYRK4* (nearby cg06270401) is from the family of dual specificity tyrosine phosphorylation regulated kinases and may be involved in neuronal differentiation (90). How this gene may relate to AUD awaits further exploration.

The current findings warrant independent replication. While this is not achievable in the current pilot study, we compared our longitudinal exercise data with Zeng et al. (46), as well as, compared our drinking-related methylation sites with those of the largest epigenome-wide study by Liu et al. (48). For the comparison of exercise-related methylation changes, we were only able to examine the genes reported in Zeng et al., and overall our data appear to largely concur with their reports. For the comparison of drinking-related methylation changes, only 15 out of 906 drinking-related CpG sites identified in the current study overlapped with those reported in Liu et al. This may be partly due to that only CpG sites showing drinking associations with *p* < 1 × 10^−4^ were reported in Liu et al. Thus, along with how drinking association was identified in our study, the overlapping sites indeed met a criterion more stringent than normal independent replication. It remains a question whether any of the 906 identified CpG sites present moderate drinking association in Liu et al. as this was not reported. The fact that 14 out of the 15 overlapping sites showed consistent directions of effect between two studies appears to lend some support for the validity of the current association analysis. However, these comparisons are more of a sanity check than a validation. Due to data availability, we are not able to examine further the generalizability of the findings and thus cannot completely rule out false positives at this stage.

Also the current findings need to be interpreted in light of the following limitations. First, the current work employed an SD threshold to guard against unknown experimental variance, and a number of *a priori* methylation sites were not included for analyses. This is expected to be alleviated by more accurate sequencing techniques. Second, the discovery power was limited by the HumanMethylation27 assay that majorly covers promotor regions. And with the technique used in the current study, we were not able to discriminate between 5-methylcytosine (5 mC) and 5-hydroxymethylcytosine (5 hmC) levels. We plan to conduct a more comprehensive study using advanced techniques in the future. Third, the effect of exercise intervention on DNAm patterns needs to be verified with a more rigorous exercise level control. Fourth, in the drinking cohort, cases and controls were not matched in race. To address this issue, we included a covariate in the regression model to eliminate the confounding effects of population structure. In addition, for the highlighted findings, we further validated the observed DNAm differences associated with alcohol use in a subset of cases and controls that were further matched in race. Collectively the results suggest a low possibility that our main findings were biased by the race factor. Fifth, the current findings were obtained from DNAm of saliva. Due to the tissue-specific nature of DNAm, it remains a question to what extent these findings reflect epigenetic patterns in neurons which more directly affect neural functions. Further inspection on cross-tissue effects of the identified sites is warranted. Sixth, the current study does not provide any elucidation of causal relationships. We speculate that DNAm may present duality of predisposition and response, such that its influence on drinking behavior may be depicted by a feedback loop model. Animal studies and longitudinal high-risk studies will be needed to address this complexity.

In summary, our findings provide preliminary evidence that exercise may induce DNAm alterations opposite to those observed in alcohol drinkers vs. non-drinkers. The identified methylation sites are annotated to genes that are enriched in signaling pathways related to synaptic plasticity and endocannabinoid system, motivating further hypothesis-driven tests. Two of the identified methylation sites, though profiled in saliva, show association with impaired control over drinking, suggesting relevance with neural function and lending support for peripheral DNAm serving as biomarkers for symptom assessment and treatment. These findings hold promise for a possible molecular mechanism through which exercise may help alleviate AUD and encourage further investigation to better characterize epigenetic biomarkers for prevention and intervention of AUD.

## Author contributions

JC, VC, and JL designed research. JC conducted analyses and wrote the paper. The remaining authors contributed to the recruitment, data collection, and preprocessing. All authors critically reviewed content and approved final version for publication.

### Conflict of interest statement

The authors declare that the research was conducted in the absence of any commercial or financial relationships that could be construed as a potential conflict of interest.
