# Supplementary material for: Opposite Epigenetic Associations With Alcohol Use and Exercise Intervention
Source: Front Psychiatry. 2018 Nov 15;9:594. doi: 10.3389/fpsyt.2018.00594 (PMC6249510; doi:10.3389/fpsyt.2018.00594)
Supplement: Supplementary file 1 [file Data_Sheet_1.docx]

# Comparison of DNAm change in exercise

For a sanity check, we compared our longitudinal exercise data with a previous work by Zeng et al. [[1](#_ENREF_1)]. While most longitudinal exercise studies focused on adipose or muscle tissues [[2](#_ENREF_2)], Zeng et al. investigated the effects of 6-month moderate exercise on DNAm of blood in a breast cancer population using the Illumina Methylation27 array, which was expected to be more suitable for comparison with our data in the sense of using also peripheral tissue and the same methylation array. Zeng et al. did not provide the complete results for all the CpG sites covered by the array. Instead, only the top 43 genes where at least one CpG site showed a significant DNAm alteration were reported (Table 1 in [[1](#_ENREF_1)]). We then examined in our data how the DNAm patterns changed at these 43 CpG sites. To compare as many of the 43 genes as we could, we included 27,490 CpG sites in the analysis without imposing the standard deviation threshold. We then conducted paired t-test (baseline versus follow-up) on 53 participants. CpG sites presenting significant DNAm alterations due to exercise were then determined based on p < 0.05 (false discovery rate controlled for 27,490 sites). Subsequently, genes annotated to these identified CpG sites were summarized. A total of 24 out of the 43 genes had CpG sites presenting genome-wide significant DNAm changes in our data. We then compared the directions of DNAm changes in two studies, as summarized in Table S1. Among the 24 genes, 15 showed DNAm changes consistent with those reported in Zeng et al. (highlighted in red in Table S1). Another 5 of the 24 genes had underlying CpG sites presenting both positive and negative DNAm changes (highlighted in yellow), for which we could not determine if our observations echoed those of Zeng et al. as the latter did not provide CpG information. The remaining 4 of the 24 genes showed inconsistent DNAm changes between the two studies (highlighted in red). Overall our data appear to largely concur with Zeng et al.. However, this is more of a sanity check than a validation. Due to data availability, currently we are not able to examine further the generalizability of the findings.

# Correlation between cg10007262 and cg00542846

The DNA methylation (DNAm) levels at these two CpG sites are highly correlated in the current data. The observed correlations are as follows: r = 0.77 (p = 1.11×10^-16^) in 81 healthy controls; r = 0.78 (p = 8.01×10^-18^) in 81 drinkers; r = 0.79 (p = 1.59×10^-12^) in 53 healthy participants’ baseline profiles; r = 0.73 (p = 4.65×10^-10^) in 53 healthy participants’ follow-up profiles.

# Table S1: Comparison of DNAm changes in exercise: our data versus Zeng et al. [[1](#_ENREF_1)].

| **Zeng et al.** | | | | |  | **Our data** | | | |
| --- | --- | --- | --- | --- | --- | --- | --- | --- | --- |
| **Gene** | **Exercise group  DNAm change** | **Usual care group  DNAm change** | **Difference  between groups** | **P-value** |  | **Gene** | **CpG site** | **DNAm change** | **P-value** |
| EPS15 | 0.0175 | -0.0121 | 0.0296 | 1.27E-07 |  | EPS15 | cg14407667 | 0.0076 | 2.58E-03 |
| KIAA0980 | -0.0036 | 0.0157 | -0.0193 | 4.68E-06 |  | KIAA0980 | cg09088834 cg17729667 | -0.0263 0.0181 | 2.72E-07 4.19E-12 |
| PLAGL1 | -0.0600 | 0.0154 | -0.0754 | 6.77E-06 |  | PLAGL1 | cg08263357 cg14161241 cg17895149 cg22378065 cg25350411 | 0.0156 -0.0208 -0.02178 0.0190 -0.0202 | 5.57E-03 1.67E-03 1.89E-03 1.62E-03 9.03E-03 |
| MEG3 | -0.0072 | 0.0256 | -0.0328 | 7.93E-06 |  | MEG3 | cg05711886 cg15101633 | -0.0133 0.0126 | 3.47E-03 9.36E-03 |
| DYDC1 | 0.0127 | -0.0233 | 0.0360 | 9.40E-06 |  | DYDC1 | cg18396533 | -0.0471 | 2.18E-07 |
| WNT7A | 0.0117 | 0.0036 | 0.0081 | 9.55E-06 |  | WNT7A | cg00625653 | -0.0077 | 7.79E-07 |
| SULF1 | 0.0172 | -0.0010 | 0.0182 | 9.55E-06 |  | SULF1 | cg02283643 | 0.0120 | 1.30E-03 |
| AQP5 | 0.0214 | -0.0055 | 0.0269 | 9.72E-06 |  | AQP5 | cg19220825 | 0.0361 | 4.66E-03 |
| ORM2 | -0.0233 | 0.0006 | -0.0239 | 1.22E-05 |  | ORM2 | cg16408565 | -0.0144 | 3.55E-09 |
| C1R | 0.0125 | 0.0010 | 0.0115 | 1.26E-05 |  | C1R | cg15776355 | -0.0367 | 3.13E-04 |
| GAB1 | -0.0369 | 0.0080 | -0.0450 | 1.53E-05 |  | GAB1 | cg01601573 | -0.0241 | 2.41E-04 |
| INSRR | 0.0033 | -0.0395 | 0.0428 | 1.63E-05 |  | INSRR | cg12995941 | 0.0303 | 2.35E-05 |
| CDC26 | 0.0200 | 0.0015 | 0.0185 | 1.78E-05 |  | CDC26 | cg11233228 | 0.0097 | 6.45E-07 |
| TMEM100 | 0.0132 | -0.0104 | 0.0237 | 2.32E-05 |  | TMEM100 | cg19403377 | -0.0321 | 3.32E-05 |
| LRRC14 | -0.0257 | -0.0099 | -0.0158 | 2.41E-05 |  | LRRC14 | cg21226234 | -0.0141 | 2.57E-03 |
| C8orf53 | 0.0172 | 0.0005 | 0.0167 | 2.86E-05 |  | C8orf53 | cg24971490 | 0.0044 | 2.51E-02 |
| MSX1 | -0.0202 | 0.0275 | -0.0476 | 3.47E-05 |  | MSX1 | cg06677140 cg09748975 cg26615830 cg27038439 | -0.0125 -0.0528 0.0037 -0.0236 | 4.03E-07 5.85E-10 5.48E-03 2.17E-05 |
| HINT2 | 0.0097 | 0.0032 | 0.0065 | 3.59E-05 |  | HINT2 | cg01328164 | 0.0030 | 5.95E-03 |
| PCTK3 | -0.0043 | 0.0202 | -0.0245 | 3.61E-05 |  | PCTK3 | cg06948937 cg07713493 | -0.0491 -0.0354 | 3.91E-05 1.96E-07 |
| WNK3 | -0.0127 | 0.0377 | -0.0503 | 3.79E-05 |  | WNK3 | cg12707233 | -0.0193 | 7.50E-04 |
| OSTF1 | 0.0118 | -0.0041 | 0.0159 | 4.01E-05 |  | OSTF1 | cg08625022 | 0.0153 | 1.77E-07 |
| ERVK6 | -0.0032 | -0.0300 | 0.0268 | 4.28E-05 |  | ERVK6 | cg21349901 | -0.0061 | 3.15E-02 |
| DC-UbP | 0.0140 | 0.0005 | 0.0135 | 4.47E-05 |  | DC-UbP | cg00076645 | 0.0039 | 4.24E-02 |
| DCC | 0.0512 | 0.0133 | 0.0379 | 4.89E-05 |  | DCC | cg02624705 cg18572014 cg18801691 | 0.0179 0.0131 -0.0174 | 7.10E-05 4.64E-04 3.85E-04 |

*Green: genes with consistent DNAm changes between two studies (all the underlying CpG sites presented the same direction of changes in our data, which is consistent with the results of Zeng et al.); Yellow: genes with underlying CpG sites presenting both positive and negative DNAm changes in our data, for which we cannot determine if our observations echo those of Zeng et al. as the latter does not provide CpG information; Red: genes with inconsistent DNAm changes between the two studies (all the underlying CpG sites presented the same direction of changes in our data, which is inconsistent with the results of Zeng et al.).

# Table S2: Comparison between the drinking-related CpG sites identified in the current study and the CpG sites identified as associated with alcohol intake in the largest epigenome-wide association study by Liu et al..

| **ID** | **Chr** | **Position** |  | **Current Study** | |  | **Liu et al.** | |
| --- | --- | --- | --- | --- | --- | --- | --- | --- |
|  |  |  |  | **Beta** | **P-value** |  | **Beta** | **P-value** |
|  |  |  |  |  |  |  | **Continuous alcohol phenotype** | |
|  |  |  |  |  |  |  | **Table_S5** | |
| cg24642820 | 3 | 13462465 |  | -0.076756 | 1.68E-14 |  | -0.000335 | 1.18E-07 |
| cg18216249 | 12 | 48100805 |  | -0.160599 | 2.60E-54 |  | -0.000220 | 6.67E-08 |
| cg08529529 | 13 | 31309799 |  | -0.148657 | 1.34E-27 |  | -0.000148 | 8.02E-06 |
| cg11700584 | 14 | 50088544 |  | -0.062639 | 5.12E-09 |  | -0.000277 | 9.26E-08 |
| cg26825412 | 20 | 62681428 |  | -0.076731 | 1.45E-11 |  | -0.000178 | 1.17E-04 |
| cg07886712 | 22 | 29665413 |  | -0.094393 | 5.47E-26 |  | -0.000095 | 2.75E-04 |
|  |  |  |  |  |  |  | **Table_S6** | |
| cg09554443 | 1 | 167487762 |  | 0.067429 | 8.61E-06 |  | 0.000039 | 7.30E-03 |
| cg18216249 | 12 | 48100805 |  | -0.160599 | 2.60E-54 |  | -0.000021 | 3.37E-02 |
| cg08529529 | 13 | 31309799 |  | -0.148657 | 1.34E-27 |  | -0.000030 | 2.10E-04 |
| cg11700584 | 14 | 50088544 |  | -0.062639 | 5.12E-09 |  | -0.000016 | 2.79E-01 |
| *cg00687674 | 15 | 69373260 |  | -0.060522 | 6.06E-11 |  | 0.000058 | 4.41E-03 |
| cg02990033 | 20 | 3643863 |  | -0.051168 | 9.16E-06 |  | -0.000033 | 9.88E-03 |
|  |  |  |  |  |  |  | **Table_S7** | |
| cg00995520 | 1 | 111218282 |  | -0.057847 | 9.27E-07 |  | -0.000438 | 1.37E-02 |
| cg03483626 | 1 | 111218276 |  | -0.133674 | 9.44E-27 |  | -0.000367 | 1.23E-02 |
|  |  |  |  |  |  |  | **Table_S28** | |
| cg18216249 | 12 | 48100805 |  | -0.160599 | 2.60E-54 |  | -0.000175 | 1.04E-05 |
| cg08529529 | 13 | 31309799 |  | -0.148657 | 1.34E-27 |  | -0.000095 | 1.74E-04 |
| cg11700584 | 14 | 50088544 |  | -0.062639 | 5.12E-09 |  | -0.000202 | 4.57E-04 |
| *cg00687674 | 15 | 69373260 |  | -0.060522 | 6.06E-11 |  | 0.000066 | 3.67E-04 |
| cg02990033 | 20 | 3643863 |  | -0.051168 | 9.16E-06 |  | -0.000038 | 1.72E-03 |
|  |  |  |  |  |  |  | **Categorical alcohol phenotype** | |
|  |  |  |  |  |  |  | **Table_S12** | |
| cg26825412 | 20 | 62681428 |  | -0.076731 | 1.45E-11 |  | -0.004501 | 1.61E-06 |
|  |  |  |  |  |  |  | **Table_S13** | |
| cg00582628 | 8 | 54764515 |  | -0.114758 | 3.35E-23 |  | -0.010894 | 8.67E-05 |
| cg08529529 | 13 | 31309799 |  | -0.148657 | 1.34E-27 |  | -0.009688 | 5.14E-07 |
| cg07525077 | 14 | 21359943 |  | -0.078167 | 1.65E-08 |  | -0.014688 | 3.08E-05 |
| cg11700584 | 14 | 50088544 |  | -0.062639 | 5.12E-09 |  | -0.016198 | 1.99E-06 |
| cg02990033 | 20 | 3643863 |  | -0.051168 | 9.16E-06 |  | -0.013509 | 1.22E-05 |
|  |  |  |  |  |  |  | **Table_S14** | |
| cg00995520 | 1 | 111218282 |  | -0.057847 | 9.27E-07 |  | -0.060940 | 5.91E-06 |
| cg03483626 | 1 | 111218276 |  | -0.133674 | 9.44E-27 |  | -0.049891 | 7.32E-06 |
|  |  |  |  |  |  |  | **Table_S29** | |
| cg20870362 | 9 | 36169175 |  | -0.099302 | 1.53E-20 |  | -0.004714 | 9.78E-05 |
| cg11700584 | 14 | 50088544 |  | -0.062639 | 5.12E-09 |  | -0.014252 | 8.43E-05 |
| cg16731240 | 19 | 52391250 |  | -0.115571 | 3.60E-37 |  | -0.007997 | 1.36E-05 |

*Inconsistent directions of effect between the two studies.

# Table S3: Official full names and brief NCBI summaries of the annotated genes of the 16 CpG sites.

| **CpG site** | **Gene** | **Official full name** | **NCBI summary** |
| --- | --- | --- | --- |
| cg00510787 | C6orf96 (RMND1) | required for meiotic nuclear division 1 homolog | The protein encoded by this gene belongs to the evolutionary conserved sif2 family of proteins that share the DUF155 domain in common. This protein is thought to be localized in the mitochondria and involved in mitochondrial translation. Mutations in this gene are associated with combined oxidative phosphorylation deficiency-11. |
| cg00542846 | APP | amyloid beta precursor protein | This gene encodes a cell surface receptor and transmembrane precursor protein that is cleaved by secretases to form a number of peptides. Some of these peptides are secreted and can bind to the acetyltransferase complex APBB1/TIP60 to promote transcriptional activation, while others form the protein basis of the amyloid plaques found in the brains of patients with Alzheimer disease. In addition, two of the peptides are antimicrobial peptides, having been shown to have bacteriocidal and antifungal activities. Mutations in this gene have been implicated in autosomal dominant Alzheimer disease and cerebroarterial amyloidosis (cerebral amyloid angiopathy). |
| cg06270401 | DYRK4 | dual specificity tyrosine phosphorylation regulated kinase 4 | This gene encodes an enzyme that belongs to a conserved family of serine/threonine protein kinases. Members of this dual specificity kinase family are thought to function in the regulation of cell differentiation and proliferation, survival, and in development. |
| cg06415153 | PITPNM2 | phosphatidylinositol transfer protein membrane associated 2 | PITPNM2 belongs to a family of membrane-associated phosphatidylinositol transfer domain-containing proteins that share homology with the Drosophila retinal degeneration B (rdgB) protein |
| cg07031532 | OAZ2 | ornithine decarboxylase antizyme 2 | The protein encoded by this gene belongs to the ornithine decarboxylase antizyme family, which plays a role in cell growth and proliferation by regulating intracellular polyamines. Expression of antizymes requires +1 ribosomal frameshifting, which is enhanced by high levels of polyamines. Antizymes in turn bind to and inhibit ornithine decarboxylase (ODC), the key enzyme in polyamine biosynthesis; thus, completing the auto-regulatory circuit. This gene encodes antizyme 2, the second member of the antizyme family. Like antizyme 1, antizyme 2 has broad tissue distribution, inhibits ODC activity and polyamine uptake, and stimulates ODC degradation in vivo; however, it fails to promote ODC degradation in vitro. Antizyme 2 is expressed at lower levels than antizyme 1, but is evolutionary more conserved, suggesting it likely has an important biological role. Studies also show different subcellular localization of antizymes 1 and 2, indicating specific function for each antizyme in discrete compartments of the cell. |
| cg12286890 | XCL2 | X-C motif chemokine ligand 2 | N/A |
| cg12671744 | FAAH | fatty acid amide hydrolase | This gene encodes a protein that is responsible for the hydrolysis of a number of primary and secondary fatty acid amides, including the neuromodulatory compounds anandamide and oleamide |
| cg14760714 | RPUSD2 | RNA pseudouridylate synthase domain containing 2 | N/A |
| cg15364618 | CIDEB | cell death-inducing DFFA-like effector b | N/A |
| cg15679651 | MAP4K1 | mitogen-activated protein kinase kinase kinase kinase 1 | N/A |
| cg17091851 | LOC348174 (CLEC18A) | C-type lectin domain family 18 member A | This is one of three closely related paralogous genes on chromosome 16 encoding secreted proteins containing C-type lectin domains. These domains bind to carbohydrates in the presence of calcium, and may be involved in cell adhesion, immune response and apoptosis. |
| cg18241160 | CDC2L2 (CDK11A) | cyclin dependent kinase 11A | This gene encodes a member of the serine/threonine protein kinase family. Members of this kinase family are known to be essential for eukaryotic cell cycle control. Due to a segmental duplication, this gene shares very high sequence identity with a neighboring gene. These two genes are frequently deleted or altered in neuroblastoma. The protein kinase encoded by this gene can be cleaved by caspases and may play a role in cell apoptosis. |
| cg24648715 | TCEAL3 | transcription elongation factor A like 3 | This gene encodes a member of the transcription elongation factor A (SII)-like (TCEAL) gene family. Members of this family contain TFA domains and may function as nuclear phosphoproteins that modulate transcription in a promoter context-dependent manner. Multiple family members are located on the X chromosome |
| cg24792360 | FUCA1 | alpha-L-fucosidase 1 | The protein encoded by this gene is a lysosomal enzyme involved in the degradation of fucose-containing glycoproteins and glycolipids. Mutations in this gene are associated with fucosidosis (FUCA1D), which is an autosomal recessive lysosomal storage disease |
| cg26825412 | SOX18 | SRY-box 18 | This gene encodes a member of the SOX (SRY-related HMG-box) family of transcription factors involved in the regulation of embryonic development and in the determination of the cell fate. The encoded protein may act as a transcriptional regulator after forming a protein complex with other proteins. This protein plays a role in hair, blood vessel, and lymphatic vessel development. Mutations in this gene have been associated with recessive and dominant forms of hypotrichosis-lymphedema-telangiectasia. |

# Table S4: Comparison of test statistics of DNAm differences in alcohol use at the 15 identified CpG sites: with and without considering cell type proportions as covariates.

| CpG_site | Without covariates of cell type proportions | |  | With covariates of cell type proportions | |
| --- | --- | --- | --- | --- | --- |
|  | P-value | T-value |  | P-value | T-value |
| cg00510787 | 7.59E-06 | -4.6342 |  | 9.39E-06 | -4.5857 |
| cg00542846 | 5.71E-09 | -6.1721 |  | 5.96E-09 | -6.1682 |
| cg06270401 | 8.05E-06 | -4.6201 |  | 3.68E-08 | -5.8022 |
| cg06415153 | 1.37E-11 | -7.3082 |  | 2.22E-16 | -9.2047 |
| cg07031532 | 4.00E-08 | 5.7810 |  | 2.38E-08 | 5.8913 |
| cg12286890 | 2.74E-09 | 6.3158 |  | 2.73E-09 | 6.3219 |
| cg12671744 | 2.33E-06 | 4.9073 |  | 3.16E-08 | 5.8331 |
| cg14760714 | 4.09E-09 | -6.2376 |  | 3.30E-09 | -6.2849 |
| cg15364618 | 1.27E-07 | 5.5410 |  | 7.31E-08 | 5.6605 |
| cg15679651 | 2.15E-07 | -5.4306 |  | 1.04E-10 | -6.9443 |
| cg17091851 | 5.66E-06 | 4.7028 |  | 3.71E-06 | 4.8033 |
| cg18241160 | 2.38E-12 | -7.6226 |  | 1.68E-12 | -7.6936 |
| cg24648715 | 1.65E-13 | -8.0913 |  | 1.09E-13 | -8.1732 |
| cg24792360 | 1.00E-07 | 5.5912 |  | 1.14E-07 | 5.5670 |
| cg26825412 | 1.45E-11 | -7.2978 |  | 1.89E-14 | -8.4755 |

**Table S5: Comparison of test statistics of DNAm differences in exercise at the 15 identified CpG sites: with and without considering cell type proportions as covariates.**

| CpG_site | Without covariates of cell type proportions | |  | With covariates of cell type proportions | |
| --- | --- | --- | --- | --- | --- |
|  | P-value | T-value |  | P-value | T-value |
| cg00510787 | 3.65E-06 | 4.8922 |  | 6.25E-06 | 4.7660 |
| cg00542846 | 4.65E-06 | 4.8336 |  | 1.32E-05 | 4.5796 |
| cg06270401 | 7.65E-05 | 4.1191 |  | 8.50E-07 | 5.2453 |
| cg06415153 | 3.10E-07 | 5.4714 |  | 6.67E-13 | 8.2238 |
| cg07031532 | 1.01E-07 | -5.7256 |  | 4.25E-07 | -5.4061 |
| cg12286890 | 1.78E-06 | -5.0653 |  | 5.26E-06 | -4.8086 |
| cg12671744 | 2.71E-05 | -4.3917 |  | 2.17E-07 | -5.5608 |
| cg14760714 | 1.41E-06 | 5.1195 |  | 2.72E-06 | 4.9686 |
| cg15364618 | 3.08E-02 | -2.1890 |  | 1.81E-02 | -2.4013 |
| cg15679651 | 3.26E-05 | 4.3438 |  | 3.14E-09 | 6.4904 |
| cg17091851 | 4.26E-04 | -3.6402 |  | 1.78E-04 | -3.8908 |
| cg18241160 | 6.43E-07 | 5.3034 |  | 1.85E-06 | 5.0611 |
| cg24648715 | 4.55E-05 | 4.2570 |  | 1.39E-04 | 3.9602 |
| cg24792360 | 7.41E-03 | -2.7314 |  | 2.17E-02 | -2.3308 |
| cg26825412 | 3.08E-05 | 4.3591 |  | 8.43E-07 | 5.2472 |

*The p-values in the second column (without covariates of cell type proportions) were not the same as those reported in Table 3 (the last column), because the latter used paired t-test while the p-values reported here were obtained with regression for a fair comparison with those obtained with covariates of cell type proportions.

# Table S6: DNAm differences of the 15 highlighted CpG sites in 29 exercise participants who increased their VO_2_ max (maximal oxygen uptake) at follow-up.

| CpG_site | Gene | Mean_Baseline | Mean_Followup | P-value |
| --- | --- | --- | --- | --- |
| cg00510787 | C6orf96 | 0.2380 | 0.2916 | 1.54E-02 |
| cg00542846 | APP | 0.1664 | 0.2025 | 1.64E-02 |
| cg06270401 | DYRK4 | 0.4532 | 0.5319 | 8.73E-05 |
| cg06415153 | PITPNM2 | 0.4363 | 0.5183 | 1.48E-05 |
| cg07031532 | OAZ2 | 0.1423 | 0.0769 | 8.89E-05 |
| cg12286890 | XCL2 | 0.6325 | 0.5092 | 1.31E-06 |
| cg12671744 | FAAH | 0.2777 | 0.1913 | 9.24E-06 |
| cg14760714 | RPUSD2 | 0.1499 | 0.2010 | 2.43E-04 |
| cg15364618 | CIDEB | 0.2861 | 0.2422 | 6.78E-04 |
| cg15679651 | MAP4K1 | 0.3439 | 0.4279 | 1.90E-05 |
| cg17091851 | LOC348174 | 0.5252 | 0.4639 | 7.81E-05 |
| cg18241160 | CDC2L2 | 0.4099 | 0.5254 | 1.27E-03 |
| cg24648715 | TCEAL3 | 0.2766 | 0.3151 | 2.34E-07 |
| cg24792360 | FUCA1 | 0.5224 | 0.4779 | 1.99E-05 |
| cg26825412 | SOX18 | 0.6652 | 0.7202 | 1.28E-04 |

# Table S7: DNAm differences of the 15 identified CpG sites in 52 drinkers and 52 controls matched in age, sex and race.

| CpG_site | Gene | Mean_Control | Mean_Drinker | P-value |
| --- | --- | --- | --- | --- |
| cg00510787 | C6orf96 | 0.2649 | 0.16108 | 1.19E-06 |
| cg00542846 | APP | 0.2399 | 0.13803 | 3.08E-09 |
| cg06270401 | DYRK4 | 0.4895 | 0.40699 | 4.18E-07 |
| cg06415153 | PITPNM2 | 0.5174 | 0.41939 | 3.89E-13 |
| cg07031532 | OAZ2 | 0.1018 | 0.155 | 1.52E-04 |
| cg12286890 | XCL2 | 0.6142 | 0.72247 | 1.73E-07 |
| cg12671744 | FAAH | 0.2200 | 0.29795 | 2.43E-06 |
| cg14760714 | RPUSD2 | 0.2327 | 0.13725 | 5.34E-09 |
| cg15364618 | CIDEB | 0.1969 | 0.31884 | 3.43E-07 |
| cg15679651 | MAP4K1 | 0.3170 | 0.24649 | 9.46E-09 |
| cg17091851 | LOC348174 | 0.4793 | 0.53419 | 4.09E-04 |
| cg18241160 | CDC2L2 | 0.5951 | 0.33696 | 4.35E-13 |
| cg24648715 | TCEAL3 | 0.3401 | 0.25481 | 1.43E-09 |
| cg24792360 | FUCA1 | 0.4665 | 0.53719 | 1.17E-06 |
| cg26825412 | SOX18 | 0.6827 | 0.60283 | 6.42E-10 |

References:

1. Zeng HM, Irwin ML, Lu LG, Risch H, Mayne S, Mu LN, Deng Q, Scarampi L, Mitidieri M, Katsaros D *et al*: **Physical activity and breast cancer survival: an epigenetic link through reduced methylation of a tumor suppressor gene L3MBTL1**. *Breast Cancer Res Tr* 2012, **133**(1):127-135.

2. Voisin S, Eynon N, Yan X, Bishop DJ: **Exercise training and DNA methylation in humans**. *Acta Physiol* 2015, **213**(1):39-59.
